# A multi-function glass shield for neutrons and gamma rays of boron- and bismuth-reinforced silicate glass

**DOI:** 10.1038/s41598-024-73977-6

**Published:** 2024-10-18

**Authors:** Hadeer M. Nasr El Din, Aly Saeed, Eman Salem, R. M. El Shazly, Magda Abdel Wahab

**Affiliations:** 1 Basic Science Department, Modern Academy for Engineering and Technology, Cairo, Egypt; 2 Mathematical and Natural Science Department, Faculty of Engineering, Egyptian Russian University, Badr, 11829 Cairo Egypt; 3 Physics Department, Faculty of Womens for Arts, Science, and Educations, Ain Shams University, Cairo, Egypt; 4 Physics Department, Faculty of Science, Al-Azhar University, Nasr City, 11884 Cairo Egypt

**Keywords:** Shielding materials, Silicate glass, Structural, Thermal, Mechanical, And optical properties, Applied physics, Nuclear physics, Optical physics

## Abstract

A successful attempt to produce a multi-function glass shield for attenuating neutrons and gamma rays by reinforcing a silicate glass network with boron and bismuth has been accomplished. A composition of 20SiO_2_-80Na_2_O (BSiBi0) was proposed to be used as a host glass network and prepared using the melt/annealing techniques. The low concentration of SiO_2_ in BSiBi0 was not sufficient to form a stable glass network. Then, the proposed BSiBi0 was modified with 10, 20, 30, and 40 mol% of each of B_2_O_3_ and Bi_2_O_3_ (BSiBi1, BSiBi2, BSiBi3, and BSiBi4) simultaneously. The structural effects of adding B^3+^ and Bi^3+^ were studied through X-ray diffraction, density, and FTIR, which all showed enhancement of glass forming ability, a former role of Bi^3+^ ions, and crowded the glass network by BO_4_ units. The derived structural parameters $-$ molar volume, mean silicon – silicon separation, mean boron – boron separation, oxygen packing density, packing density, and number of bridging/non-bridging oxygen $-$ were extensively discussed to explore the impact of B^3+^ and Bi^3+^ on the formed network. The richness of the proposed host glass network by B^3+^ and Bi^3+^ enhanced its thermal stability. The obtained elastic properties by ultrasonic measurements reflect the increase of the glass rigidity with increasing concentrations of B^3+^ and Bi^3+^ ions. The obtained glasses have high visible light transparency and almost complete UV absorption. The measured shielding parameters against two types of neutron energies (total slow and slow) and a wide range of gamma rays’ energies showed a significant improvement in the shielding efficiency of the considered glasses. The total slow neutrons, slow neutrons, and gamma rays’ attenuation abilities were improved by 22.9, 135.5, and 73.8 $-$ 199.5%. High thermal stability, elasticity, visible light transparency, and neutrons and gamma rays’ attenuation performance features give the produced glasses, especially BSiBi4 glass, preference as shielding materials in nuclear fields.

## Introduction

Intensive studies are continuing the different glass networks such as silicate, borate, phosphate, etc. because of their great significance in a large number of optical, electronic, nuclear, medical and other applications ^1–3^. These studies aim to enrich the existing glass properties and add more properties to it ^1–3^. One of the rare paths of these studies is the reinforcing of the glass network by more than one ion of two or more chemically different groups simultaneously. Silicate glass network is one of the most popular glasses being its availability and cheapest ^3,4^. They boast excellent mechanical strength, and optical transparency, and exhibit high chemical and thermal stability ^3,4^. The unmixed silicate glass network consists of a three-dimensional random network across the interconnection of SiO_4_ tetrahedral with each other through bridging oxygen ^3,4^. The inserted additives from various chemical groups, such as alkali ions (e.g., Li^+^, Na^+^, and K^+^), transition metal ions (e.g., Zn^2+^, Fe^2+^, and Ti^2+^), and alkaline earth ions (e.g., Mg^2+^, Ca^2+^, and Ba^2+^), into the silicate network produce a set of structural changes causing its enhancement with extra properties ^3,4^. In addition to containing all the famous glass formers, silicate, borate, phosphate, etc., metalloids group ions such as B, Si, P, Ge, As, Sb, and Te enhance glass networks with many optical, mechanical, electrical, and thermal properties ^5–9^. In addition to its role as one of the famous glass formers with high stability, boron ions impart many properties when added to other glass networks such as phosphate and silicate ^2,5^. Its role as a glass former enables it to enter directly as a main former in the glass networks to which it will be added such as silicate and phosphate creating a double network such as borosilicate and borophosphate ^6,7^. The ability of B^3+^ ions to form chemical bonds with varying covalent degrees formed with oxygen ions a 3 or/and 4 coordination number within the glass network enhances the elasticity, thermal stability, and optical transparency of the various glass networks ^2,5^. The geometrical changes of the borate units from neutral trigonal planar (3-coordinated boron N_3_) to charged tetrahedral (4-coordinated boron N_4_) is the main controller in imparting varying properties to the glass network ^10,11^. On the other hand, B nucleus is considered one of the most effective absorbers for slow neutrons with lower secondary production of gamma rays ^12,13^. It also having a significant ability to attenuate of the fast neutrons as a light nucleus ^14,15^. Post-transition metals ions such as Bi^3+^, Pb^2+^, and Al^3+^ add amazing features of the glass networks ^11,16,17^. In addition, post-transition metal ions have a dual role function, modifier and former, inside the glass networks, which riches the glass structure that in turn is reflected upon its optical, mechanical, and thermal properties…etc. ^16,17^. Being a heavy post-transition metal, Bi^3+^ ion has outstanding nonlinear optical properties, which arise due to its high polarizability and small field strength of Bi^3+^^18,19^. Bi^3+^ ion has a broad emission spectrum extended from UV up to red regions according to the pumped excitation wavelength ^20^. These features make the materials-based Bi^3+^ favorable for utilization in photonics and optoelectronics devices ^18–20^. Structurally, Bi^3+^ ion has a dual role in the glass network; modifier through formation the octahedral BiO_6_ or former through formation the pyramidal BiO_3_^21^. From a nuclear point of view, as it is one of the most electron-rich and eco-friendly elements, the role of Bi has emerged as an effective attenuator of gamma rays of different energies. Recently, many attempts were made to enhance the various host materials with Bi to increase their attenuation efficiency, enabling them to be used as protective shields from gamma rays in various nuclear domains ^16,21,22^. Finally, extensive study has been conducted on strengthening different glass networks by adding single ions from the different chemical groups, such as B^3+^, Na^+^, Fe^2+^, Ni^2+^, Pb^2+^, or Bi^3+^. However, due to the complexity of examining and analyzing the results, studying the enhancement of different glass networks’ properties by adding two or more ions simultaneously is extremely rare.

Based on the above, the study aimed to improve the ability of silicate glass to attenuate neutrons and gamma rays by simultaneously incorporating B^3+^ and Bi^3+^. Given that glass shields must exhibit robust mechanical properties, excellent thermal stability, and crucially, transparency, these attributes were thoroughly investigated to assess the suitability of the developed glasses as shielding materials. Prior to these assessments, the structural properties were analyzed to understand the changes in the glass network resulting from the addition of B^3+^ and Bi^3+^ using X-ray diffraction, density measurements, and Fourier transform infrared spectra.

## Experimental procedures

A host glass matrix of composition 20SiO_2_-80Na_2_O (BSiBi0) was proposed, and attempts were made to prepare it using a melting/annealing method up to a melting temperature of 1300 °C. A series of substitutions, based on replacing sodium with bismuth and boron as listed in Table 1 to prepare a series of SiO_2_-Na_2_O-B_2_O_3_-Bi_2_O_3_ glass (BSiBi glass series) were performed. A high purity SiO_2_, Na_2_CO_3_, H_3_BO_3_, and Bi_2_C_3_O_9_ were weighted on high accurate balance to synthesis the different batches of the proposed glasses. The weighted raw materials were grinded in a porcelain mortar for 30 min to obtain a highly homogeneous mixture. The homogeneous powder of all sample was then melted at 1050 °C in platinum crucible. During the melting process, care was taken to continuously stir to get homogeneous and bubbles free molten. The molten was annealed at 320 °C in steel mold for one hour. The annealing temperature and duration were selected following a polarized light microscopy test. Initially, an annealing temperature of 300 °C for one hour was suggested to avoid excessive temperatures that could induce crystallization within the glass network. However, polarized light microscopy revealed intense thermal stress after annealing at 300 °C. Consequently, the annealing temperature was increased to 320 °C, and the test was repeated to ensure the complete removal of thermal stresses.

**Table 1:** The chemical compositions of the produced glasses.

Glass Code	Chemical composition (mol%)			
	SiO_2_	Na_2_O	B_2_O_3_	Bi_2_O_3_
BSiBi0	20	80	0	0
BSiBi1	20	60	10	10
BSiBi2	20	40	20	20
BSiBi3	20	20	30	30
BSiBi4	20	0	40	40

The structural changes resulting from the addition of B^3+^ and Bi^3+^ ions into the proposed core silicate network were studied through X-ray diffraction XRD, density, density-based parameters, and Fourier Transformation Infrared FTIR spectroscopy. The structural phase of the produced materials was identified using XRD-7000 Shimadzu X-ray diffractometer (Cu-Kα, $\lambda$ = 1.504 Å). Archimedes’ principle according to Eq. (1)^23,24^ was used to measure the glass density $\rho$, while the molar volume ${V}_{m}$ was estimated using Eq. (2)^23,24^.

1 $$\rho =\frac{{W}_{a}}{{W}_{a}-{W}_{l}}\times {\rho }_{l}$$

2 $${V}_{m}=\frac{M}{\rho }$$

where, ${W}_{a}$ and ${W}_{l}$ are the sample weight in air and liquid, ${\rho }_{l}$ is liquid density (toluene was used here, ${\rho }_{l}=0.866$ gm/cm^3^), and *M* is the molar mass.

In the spectral range 400-4000 cm^-1^ with an uncertainty $\pm 2$, a JASCO FT-IR-300 spectrophotometer was used to study the structural units of the produced glasses. Differential Scanning Calorimeter DSC measurements through a TA Instruments, SDT Q600 in an open platinum pan was used to obtain the glass transition ${T}_{g}$, onset crystallization ${T}_{c1}$ & ${T}_{c2}$, and maximum crystallization rate ${T}_{p1}$ & ${T}_{p2}$ temperatures. The DSC measurements were carried out up to 900 °C at heating rates of 5 °C/min in a high-purity nitrogen atmosphere with a flow rate of 15 Psi. Ultrasonic longitudinal ${v}_{L}$ and shear ${v}_{S}$ wave velocities were measured using pulse-echo method. Based on the measured values of ${v}_{L}$ and ${v}_{S}$, longitudinal C11, shear C44, bulk B, and Young Y moduli, Poisson’s ratio $\sigma$, microhardness H_UT_, acoustic impedance ${Z}_{i}$, and Debye temperature ${\theta }_{D}$ were estimated using the following Eqs. (^5,25^)

3 $${C}_{11}=\rho {v}_{L}^{2}$$

4 $${C}_{44}=\rho {v}_{s}^{2}$$

5 $$B=\frac{\rho \left(3{v}_{L}^{2}-4{v}_{S}^{2}\right)}{3}$$

6 $$Y=\frac{\rho {v}_{S}^{2}\left(3{v}_{L}^{2}-4{v}_{S}^{2}\right)}{{v}_{L}^{2}-{v}_{S}^{2}}$$

7 $$\sigma =\frac{\left({v}_{L}^{2}-{2v}_{S}^{2}\right)}{2\left({v}_{L}^{2}-{v}_{S}^{2}\right)}$$

8 $${H}_{UT}=\frac{\left(1-2\sigma \right)}{6\left(1+\sigma \right)}$$

9 $${Z}_{i}={v}_{mean}\rho$$

10 $${\theta }_{D}=\frac{h}{{K}_{B}}{\left(\frac{9{N}_{A}}{4\pi {V}_{m}}\right)}^{1/3}{v}_{mean}$$

where, $h$, ${K}_{B}$, ${N}_{A}$, ${V}_{m}$, and ${v}_{mean}$ are the Planck’s constant, the Boltzmann’s constant, the Avogadro’s number, molar volume, and the mean ultrasonic velocity, which calculated by

$${v}_{mean}={\left(\frac{1}{3}\left(\frac{1}{{v}_{L}^{3}}+\frac{2}{{v}_{s}^{3}}\right)\right)}^{-1/3}$$

A Jenway 6405 UV/Vis Spectrophotometer was used to measure the absorbance and transmittance spectra of the considered glasses in the spectral range 190–1100 nm with a resolution of 2 nm.

**Fig. 1:**
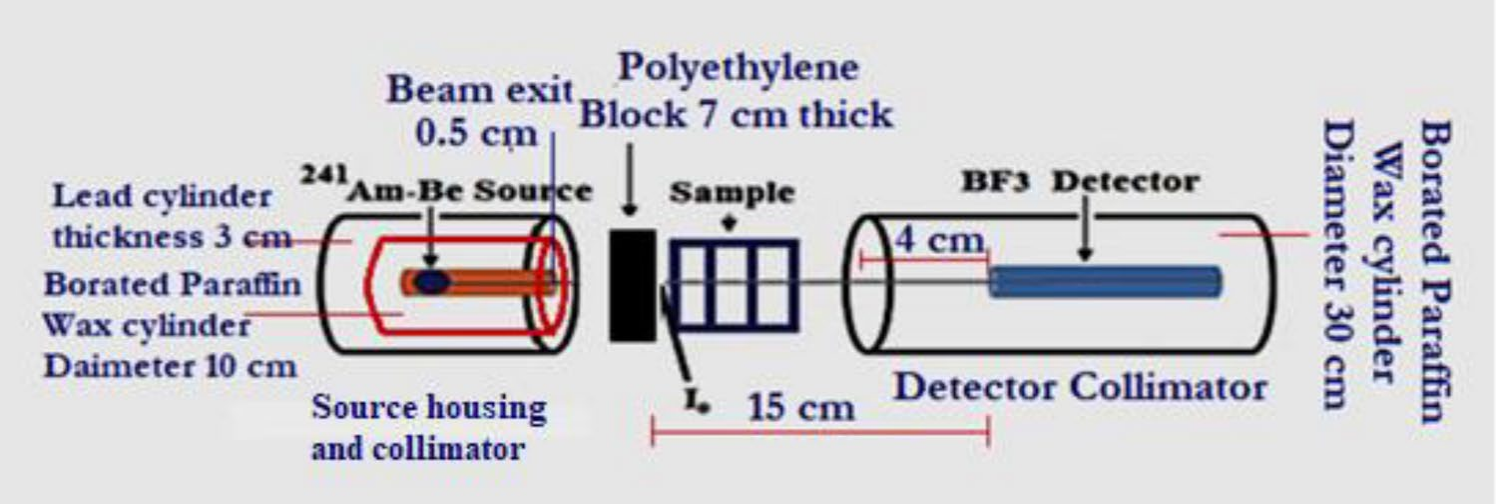
Measurement setups of the neutrons’ attenuation performance of the considered BSiBi1, BSiBi2, BSiBi3, and BSiBi4 glasses.

Examination of the attenuation ability of neutrons and gamma rays of the considered BSiBi glass series was carried out through two different neutrons energy ranges, total slow and slow, and nine gamma ray energies ranged between 121.78 and 1407.24 keV. A collimated beam of total slow and slow neutrons emitted from ^241^Am-Be neutron source, 100 mCi, and BF_3_ neutron detector were used to examine the efficiency of the considered BSiBi glass series to attenuation neutrons. The neutron spectrum of the ^241^Am–Be source is about 23% slow neutrons (neutrons with energy $< 1\text{ keV}$), and the rest are fast neutrons (up to 11 MeV) ^26,27^. In the current study, the slow neutrons concept signifies the neutrons that emerged from the source and entered the studied glass specimen with energy $<1\text{ keV}$. While the total slow neutrons concept signifies the primary slow neutrons that are emitted from the source alongside the secondary slow neutrons that have been generated due to the decelerating fast neutrons’ during the collision with the glass constituents. Here, to measure the attenuation of total slow neutrons, the varies thickness of the glass samples were directly placed in front of the collimated whole-emitted neutrons from the ^241^Am-Be source. While, in the case of the slow neutrons’ attenuation, 7 cm of polyethylene block was used to decelerate the emitted fast neutrons of ^241^Am-Be and the considered glass samples were placed directly behind the polyethylene block. Figure 1 shows the geometrical arrangement of the slow and slow total neutron measurement setups. The neutron macroscopic cross section $\Sigma$ (for both slow ${\Sigma }_{S}$ and total slow ${\Sigma }_{T}$) of the considered BSiBi1, BSiBi2, BSiBi3, and BSiBi4 glasses was deduced according to Eq. (11)^12,25^

11 $$N={N}_{o}{e}^{-\Sigma x}$$

where, ${N}_{o}$ and $N$ are the neutron (total slow or slow) intensity before and after the glass thickness $x$.

**Fig. 2:**
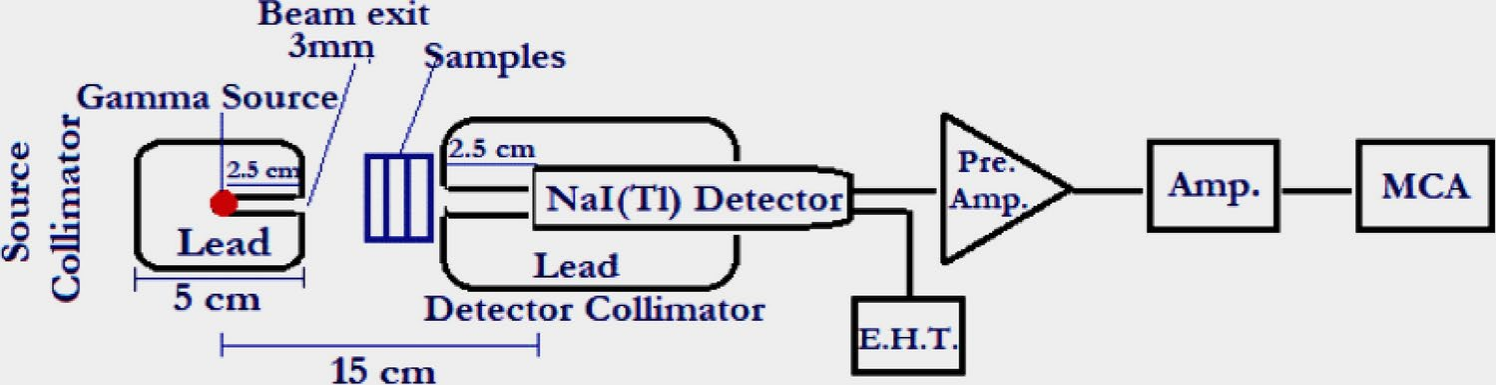
Measurement setups of the gamma rays’ attenuation performance of the considered BSiBi1, BSiBi2, BSiBi3, and BSiBi4 glasses.

Nine gamma ray energies at 121.78, 344.27, 661.64, 778.90, 964.00, 1112.40, 1173.23, 13,332.51, and 1407.24 keV emitted from ^137^Cs (9.5 $\mu$ Ci), ^60^Co (4.9 $\mu$ Ci), and ^152^Eu (3.7 $\mu$ Ci) were used to explore the attenuation ability of the fabricated BSiBi glass series. Under the setup of appropriate geometrical conditions shown in Fig. 2, which ensured the accuracy of the measurement and the lack of experimental error, the gamma rays’ attenuation measurements of the targeted BSiBi glass series were carried out. The emitted intensity of gamma rays’ energies from the used radioactive sources was measured using a NaI(Tl) detector without any glass thickness, at first, (bare beam, ${I}_{o}$). Then, the transmitted photon intensity ($I$) through the different glass thicknesses ($x$) of the studied BSiBi1, BSiBi2, BSiBi3, and BSiBi4 glasses was measured. Based on Beer-Lambert’s rule shown in Eq. (12)^16,25^, the rate of change of gamma rays’ photons intensities was plotted as a function of BSiBi glass thickness (Fig. 9 shows an example) to derive the linear attenuation coefficient ($\mu$). Furthermore, to ensure the validity of the measured results, the experimental mass attenuation coefficient (${\sigma }_{exp.}$) was estimated through Eq. (13)^16,25^ and theoretically calculated (${\sigma }_{the.}$) using the XCOM software, and the results were compared to each other. Finally, to closely examine the efficiency of the studied BSiBi glass shield, whether against neutrons or gamma rays, the half-value layer for both neutrons (${\text{HVL}}_{\text{n}}$) and gamma rays (${\text{HVL}}_{\upgamma }$) was calculated using Eq. (14)^28,29^.

12 $$I={I}_{o}{e}^{-\mu x}$$

13 $${\sigma }_{exp}=\frac{\mu }{\rho }$$

14 $${HVL}_{n}=\frac{\text{ln}2}{\Sigma } and {HVL}_{\gamma }=\frac{\text{ln}2}{\mu }$$

Finally, to overcome the experimental error in the conducted measurements, for neutrons and gamma rays, the measurements were repeated five times for 300 s of each one, and the average was taken to become the final intensity reading for both neutrons ($N$) and gamma rays ($I$). Then, the experimental errors in both neutrons’ macroscopic cross-section and gamma rays’ attenuation coefficients were calculated by the Eqs. (15 and 16)^12,16,25,28,29^.

15 $$\Delta \Sigma = \frac{1}{x}\sqrt {\left( {\frac{{\Delta N_{o} }}{{N_{o} }}} \right)^{2} + \left( {\frac{\Delta N}{N}} \right)^{2} + \left( {\ln \frac{{N_{o} }}{N}} \right)^{2} \left( {\frac{\Delta x}{x}} \right)^{2} }$$

16 $$\Delta \mu =\frac{1}{x}\sqrt{{\left(\frac{\Delta {I}_{o}}{{I}_{o}}\right)}^{2}+{\left(\frac{\Delta I}{I}\right)}^{2}+{\left(ln\frac{{I}_{o}}{I}\right)}^{2}{\left(\frac{\Delta x}{x}\right)}^{2}}$$

## Results and discussion

### Ocular examination and preparation procedures observations

**Fig. 3:**
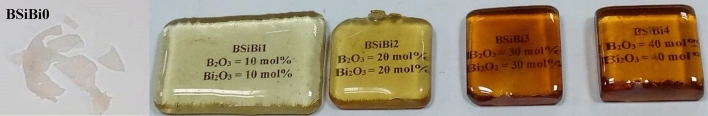
The produced BSiBi glass series.

Transparency-colored glasses were obtained of the BSiBi1, BSiBi2, BSiBi3, and BSiBi4 glasses, as shown in Fig. 3. Many facts relevant to the considered glasses based on the visual examination (Fig. 3) and the notes taken during the preparation process were observed. The poor glass forming ability of BSiBi0 arises due to the low concentration of the glass former SiO_2_ and the poverty of BSiBi0 blend of the intermediate additives, which help in the process of glass formation. Although the high concentration of sodium oxide, it was not sufficient to enrich the silicate glass formability at 20 mol% of SiO_2_. With the inclusion of B^3+^ and Bi^3+^ ions, free bubbles and high-translucency glasses were obtained. A gradation in color from transparent yellowish to transparent deep brown was observed, as shown in Fig. 3, which is attributed to the addition of Bi^3+^ ions into the network. The intensity of the brown color increases with the increase in Bi_2_O_3_ concentrations, which reflects the richness of the glass network with Bi^3+^ ions. On the other hand, no color effects arise due to the existence of B^3+^ due to B_2_O_3_ being a famous colorless glass former. Finally, it is worth mentioning that, as a result of the lack of formation of the amorphous state of the proposed host glass network BSiBi0 (which was confirmed from the visual examination, X-ray diffraction pattern, and FTIR spectrum), the thermal, mechanical, optical, and attenuation properties measurements were not carried out on it.

### Structural properties

#### X-ray diffraction

**Fig. 4:**
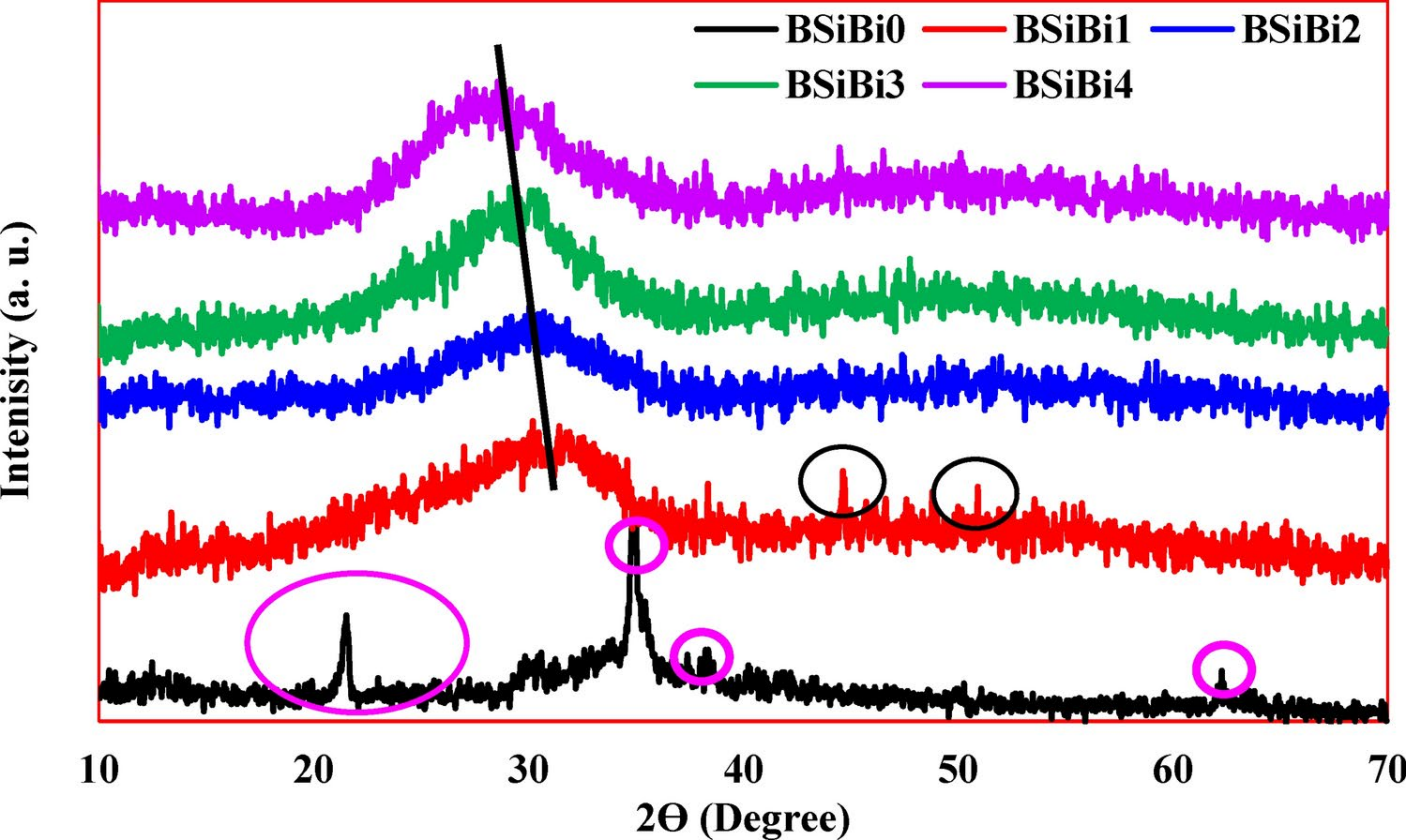
The amorphous profile of the produced materials using X-ray diffraction**.**

The analysis of the illustrated X-ray diffraction patterns in Fig. 4 strongly indicates the extent to which B^3+^ and Bi^3+^ ions enhance the glass network formability. In the proposed host material BSiBi0, the characteristic broad band of the amorphous phase did not appear; however, sharp peaks were observed at 21.56°, 34.84°, 38.40°, and 62.38°. The appeared peaks at 21.56° and 38.40° are attributed to the sodium disilicate (Na_2_Si_2_O_5_) crystal phase, while the peaks at 34.84° and 62.38° correspond to the sodium tetrasiliicate (Na_2_Si_4_O_9_) and NaOH crystalline phases. On the other hand, two very weak peaks appeared at 44.72° and 50.92° in the BSiBi1 glass sample, related to the formation of Na_2_SiO_3_ crystalline phases. From the glass point of view, the main controller in the formation of a silicate glass network is the O:Si ratio and the role of the glass modifiers. With the O:Si ratio exceeding 2.5 ^30–32^, silica glasses are difficult to form except under special circumstances, such as melting at high temperatures and rapid cooling rate. On the other hand, sodium is classified as a network modifying cation remaining as ionically bonded in the network interstices by filling them rather than becoming a part of the network, promoting the crystallization of the glass. Hence, a 20 mol% of SiO_2_ (BSiBi0) unable to form a pure amorphous phase even with a high concentration of sodium. A well-known and distinguished broad diffraction band for the formation of the amorphous phase was observed between 25.9–34.8° in the BSiBi1 glass, which contains on 20, 10, and 10 mol% of SiO_2_, B_2_O_3_, and Bi_2_O_3_. The well-definite broad diffraction band in BSiBi1 glass was conclusively attributed to the ability of B_2_O_3_ to penetrate the silicate glass network resulting in a hybrid of the borate and silicate network, borosilicate glass SiO_4_/[BO_4_/BO_3_]. Previous literature indicated that the characteristic broad network band of silicate glass usually appears in the angle range $2\theta =20-30^\circ$^33^, while that of borate glass is in the range $2\theta =20-25^\circ$^23^, which is may be clear evidence that the formed broadband in the intermediate region 25.9–34.8° is a distinctive feature of borosilicate network formation ^16,34^. However, in BSiBi1, there are two discernible weak bands at angles 44.76° and 50.92°, indicating the presence of little crystalline phases within the glass network.

The continuous shift toward lower angle 25–34°, 22.8–33.9°, and 22–33.3° is also a strong indicative of the enhancement of borosilicate network formation. Hence, as a result of the existence of SiO_4_ and augmentation of BO_4_/BO_3_ as a function in B_2_O_3_ addition; formation of SiO_4_/[BO_4_/BO_3_] reinforce the glass forming ability. On the other hand, Bi_2_O_3_ is considered a glass intermediate, which has a significant role in enhancing of glass formability. In the considered glasses, Bi_2_O_3_ enters to network as a former as appeared in density and FTIR results, reinforcing glass network formability.

#### Density and density-based parameters

Upon insertion of B_2_O_3_ and Bi_2_O_3_ ions into the proposed host glass network BSiBi0, an increase in density and molar volume was observed. The observed increase in density as shown in Table 2 is mainly attributed to the addition of Bi^3+^ ions (the densest of all constituents), which permeate the interstices formed within the studied glass network intensified its density and formed crosslinking bonds with the network, becoming part of its structure. On the other hand, three agents directly affect in the augmentation in the molar volume, which are the existence of BO_3_ units, the largest ionic radius of Bi^3+^ (1.03 Å) ^16^ compared to the other network constituents, and the formation of longer bond length $\text{Bi}-\text{O}-\text{Si}$, $\text{Bi}-\text{O}-\text{B}$, and $\text{B}-\text{Bi}-\text{Si}$ bonds. Since increasing the addition of B_2_O_3_ and Bi_2_O_3_ led to a growth in BO_4_ units compared to BO_3_ (observed from the FTIR results) in the considered BSiBi1, BSiBi3, and BSiBi4 glass networks, the two factors affecting the increase in molar volume are the ionic radius of B^3+^ and the formed longer bond length bonds. While for the BSiBi2 glass, all three factors are involved in increasing its molar volume, as the ratio of BO_3_ units exceeds the BO_4_ ones (observed in the FTIR results). The augmentation of molar volume reflects the expansion in the glass network. Mean silicon – silicon ${d}_{Si-Si}$ and mean boron—boron separation ${d}_{B-B}$ were estimated using Eqs. (17 and 18)^10,11,16^ to explore the considered network expansion.

17 $${d}_{Si-Si}={\left(\frac{{V}_{m}^{Si}}{{N}_{A}}\right)}^{1/3}$$

18 $${d}_{B-B}={\left(\frac{{V}_{m}^{B}}{{N}_{A}}\right)}^{1/3}$$

where, ${N}_{A}$ is the Avogadro’s number and ${V}_{m}^{Si}$ and ${V}_{m}^{B}$ are the volume that contains one mole of silicone and boron within the obtained network respectively, which are calculated by

$${V}_{m}^{Si}=\frac{{V}_{m}}{2\left(1-{X}_{P}\right)}$$

$${V}_{m}^{B}=\frac{{V}_{m}}{2\left(1-{X}_{P}\right)}$$

where, ${X}_{P}$ is molar fraction of SiO_2_ and B_2_O_3_.

**Table 2:** Density, density-based parameters, thermal, mechanical, and optical parameters.

Glass code					
Studied parameters		BSiBi1	BSiBi2	BSiBi3	BSiBi4
Structural Parameters	${\varvec{\rho}}$(gm/cm^3^)	3.479	4.246	4.844	5.426
	${{\varvec{V}}}_{{\varvec{m}}}$(cm^3^/mol)	29.541	33.896	38.205	41.695
	${{\varvec{d}}}_{{\varvec{S}}{\varvec{i}}-{\varvec{S}}{\varvec{i}}}$(°A)	3.943	4.128	4.296	4.423
	${{\varvec{d}}}_{{\varvec{B}}-{\varvec{B}}}$(°A)	3.792	4.128	4.492	4.869
	OPD	54.162	59.004	62.819	67.153
	PD	0.483	0.493	0.502	0.519
	N_3_	0.434	0.466	0.456	0.437
	N_4_	0.566	0.534	0.544	0.563
Thermal parameters	${{\varvec{T}}}_{{\varvec{g}}}$(°C)	341.9	303.7	340.1	405.3
	${{\varvec{T}}}_{{\varvec{c}}1}$(°C)	461.5	404.9	477.8	485.9
	${{\varvec{T}}}_{{\varvec{P}}1}$(°C)	508.4	471.4	552.7	526.2
	${{\varvec{T}}}_{{\varvec{c}}2}$(°C)	627.0	510.0	667.3	–
	${{\varvec{T}}}_{{\varvec{P}}2}$(°C)	660.7	526.7	685.9	–
	$\Delta {\varvec{T}}$(°C)	285.1	205.8	352.2	–
Mechanical parameters	${V}_{L}$(m/sec)	5279	5352	5712	6095
	${V}_{S} ($ m/sec)	2847.4	2995.6	3321.2	3522.1
	C_11_ (GPa)	96.941	121.623	158.061	201.578
	C_44_ (GPa)	28.203	38.102	53.436	67.313
	B (GPa)	59.336	70.820	86.812	111.827
	Y (GPa)	73.038	96.924	133.017	168.192
	$\sigma$(GPa)	0.295	0.272	0.245	0.249
	H_UT_ (GPa)	3.857	5.794	9.097	11.249
	${\theta }_{D}$°C	37.287	37.367	39.678	40.892
	${Z}_{i}\times {10}^{6}$(kg/m^2^.sec)	11.056	14.158	17.851	21.216
Optical parameters	${{\varvec{E}}}_{{\varvec{g}}}$(eV)	3.20	3.04	3.40	3.60
	$\Delta {\varvec{E}}$(eV)	0.345	0.357	0.328	0.322

Augmentation of both mean silicone—silicon and mean boron—boron separation as shown in Table 2 refers to the spacing of the main glass network former, silicon and boron atoms, signifying the increase of the considered borosilicate network free volume. One of the important conclusions relevant to the properties of glass materials, which is based on the analysis of molar volume and network building block atoms separation (here, ${d}_{Si-Si}$ and ${d}_{B-B}$) results, is the tightness of the glass network, which reflects the mechanical and thermal properties of the glass. The tightness of the network is accurately explored through the analysis of the behavior of oxygen packing density OPD and packing density PD, which were estimated using Eqs. (19 and 20)^10,11,16^.

19 $$OPD=\frac{\rho }{M}\times n$$

20 $$PD=\frac{\rho }{M}\sum_{i}{x}_{i}{V}_{i}$$

where, $\text{n}$ is the number of oxygen atoms per formula unit, ${x}_{i}$ the mol fraction, and ${V}_{i}$ the packing factor

$${V}_{i}=\frac{4\pi {N}_{A}}{3}\left(b{r}_{A}^{3}+c{r}_{A}^{3}\right)$$

where, ${r}_{A}$ and ${r}_{B}$ are the ionic radii of the cation and the anion, respectively.

The occurred growth in both OPD and PD was attributed to the accumulation of O^2-^ ions inside the considered glass network. The richness of the network with O^2-^ ions resulted from an increase in the concentrations of B_2_O_3_ and Bi_2_O_3_. Also, the large ionic radius of the modifier ions (here Bi^3+^ which equal 1.03 Å) compared to glass network formers (here Si^4+^ and B^3+^ which are equal 0.40 and 0.27 Å, respectively) attracts more oxygen ions leading to a growth in the interstices size, which enhance the molar volume as previously discussed. On the other hand, the formation of tetrahedral BO_4_ units in BSiBi1, BSiBi2, and BSiBi4 glasses increases packing in the glass network, which signifies tightness of the considered glasses. It was expected from the behavior of the molar volume, which indicated an increase in the free volume within the network, that the glass would be less tight, but an opposite behavior was found in both OPD and PD. On the other hand, although BSiBi2 glass contains a higher ratio of non-bridging oxygen units, the values of OPD and PD also increased, which indicates their tightness as well. the packing of BSiBi2 glass resulted from the accumulation of ions B^3+^, Bi^3+^, and O^2-^ ions within the network, and the formed $\text{Bi}-\text{O}-\text{Si}$, $\text{Bi}-\text{O}-\text{B}$, and $\text{B}-\text{Bi}-\text{Si}$ bonds. Therefore, the studied network has an expanded molar volume with tight packing inside it. The expansion of the studied glass network does not mean that there are voids inside it, as it is completely filled with Bi^3+^ ions, but the expansion of the network arose as a result of the large ionic radius of Bi^3+^ ion and the formation of longest bonds $\text{Bi}-\text{O}-\text{Si}$, $\text{Bi}-\text{O}-\text{B}$, and $\text{B}-\text{Bi}-\text{Si}$. Hence, there is no conflict between the molar volume results and glass rigidity, because there is a double effect that arises inside the considered network as a result of adding both B^3+^ and Bi^3+^ ions simultaneously.

#### FTIR

**Fig. 5:**
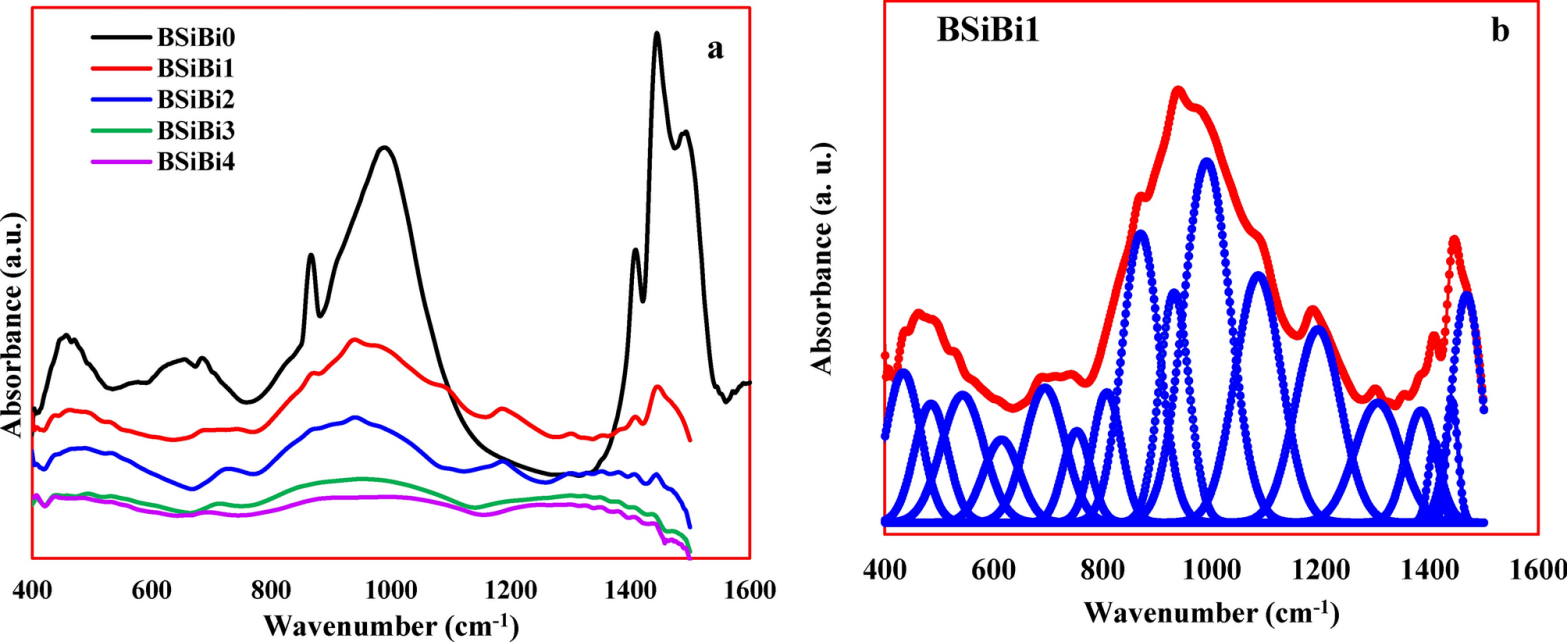
(**a**) FTIR spectra of the considered BSiBi glass series and (**b**) the deconvolution of the BSiBi1 glass.

The measured FTIR spectra along with their deconvolution (BSiBi glass as an example) are shown in Fig. 5a,b. The up normal behavior of the BSiBi0 in agreement with the XRD results, which reflected the non-amorphous phase of the BSiBi0. In the FTIR of the BSiBi1 glass samples, seventeen bands at 434, 485, 543, 614, 694, 753, 808, 870, 931, 991, 1086, 1195, 1305, 1384, 1409, 1441, and 1486 cm^−1^. This bands can be categorized as (i) metal ions bonds, here Na^+^ and Bi^3+^, (ii) silicate groups bonds, (iii) borate groups bonds, and (iv) the hybrid bonds of borosilicate network and borate-metals and silicate-metals bonds.

(i) **Metal ions bands:** The Bi–O–Bi linkages in BiO_6_ octahedral segments generated bands at 543 and 870 cm^-1^ for bending vibration and stretching vibration, respectively ^16,23^. The band at 485 cm^-1^ may be attributed to the vibrations of Na^+^^35^.
(ii) **Silicate groups bonds:** The appeared bands at 485, 991, and 1086 cm^−1^ arose as a result of bending vibration of $\text{Si}-\text{O}-\text{Si}$ and $\text{O}-\text{Si}-\text{O}$^16,36,37^, the SiO_4_ tetrahedral vibrations in the units containing two non-bridging oxygen atoms (NBOs) per silicon ^33–37^, and vibrations of Si–O-Si linkage in SiO_4_ tetrahedral units ^36,37^.
(iii) **Borate groups bonds:** the appeared bands at 614 and 694 cm^-1^ attributed to the O-B-O ^20^ and B–O–B bending vibration of triangles borate groups BO_3_^6^. The band at 753 cm^−1^ arose as a result of B_3_O–O–BO_4_ bending vibrations ^38^. The band at 808, 931, 1086, and 1195 cm^−1^ and attributed to B-O stretching in in BO_4_ tetrahedral units in various structural geometrical forms, such as di-, pyro-, and ortho-borate, etc. ^39–45^. On the other hand, the stretching vibration of B—O in BO_3_ trigonal units in various structural geometrical forms are located at 1305, 1384, and 1409 cm^−1^^43,44^. The bands at 1441 and 1486 cm^−1^ emerged due to the symmetric and asymmetric stretching of the B–O-B bond linked with three non-bridging oxygens, respectively ^43,44^.
(iv) **Hybrid bonds**: the bands at 434 may be also appeared due to the formation of $\text{B}-\text{O}-\text{Bi}$^43,44^, while that are at 485 and 694 cm^−1^ emerged due to $\text{B}-\text{O}-\text{Si}$ stretching mode ^45,46^.

Some structural changes were observed with increasing of B^3+^ and Bi^3+^ concentrations and they are shown as follows.

The variation of non-bridging (BO_3_, N_3_) and bridging oxygen (BO_4_, N_4_) units, which are brought to the glass network though adding B_2_O_3_ or impact of Bi_2_O_3_ were estimated using the following Eqs. (^10,11^) and listed in Table 2.

21 $${N}_{3}=\frac{Area \left({BO}_{3}\right)}{Area \left({BO}_{3}\right)+Area \left({BO}_{4}\right)}$$

22 $${N}_{3}=\frac{Area \left({BO}_{4}\right)}{Area \left({BO}_{3}\right)+Area \left({BO}_{4}\right)}$$

Generally, the BO_4_ units’ concertation in the studied glasses exceeds that of BO_3_ ones due to the role of Bi_2_O_3_ in the conversion of BO_3_ to BO_4_. Regarding the BSiBi2 glass, as we mentioned, the borate glass network consists mainly of non-bridging oxygen units (BO_3_ units), and its containing ratios of bridging oxygen units (BO_4_ units) arise as a result of the conversion role of the modifies to convert of BO_3_ to BO_4_. Hence, we suggest that the re-growth of the non-bridging oxygen unit resulted from the increase in bringing BO_3_ with the increase of B_2_O_3_ concentration and decelerate the conversion role of Bi_2_O_3_.

Based on the visual inspection (Fig. 3), X-ray diffraction analysis (Fig. 4), and FTIR spectra (Fig. 5), it is evident that the BSiBi0 sample did not achieve a glassy state. Consequently, measurements of its density, and thermal, mechanical, and optical properties were not feasible and are therefore not included in the results.

### Thermal properties

**Fig. 6:**
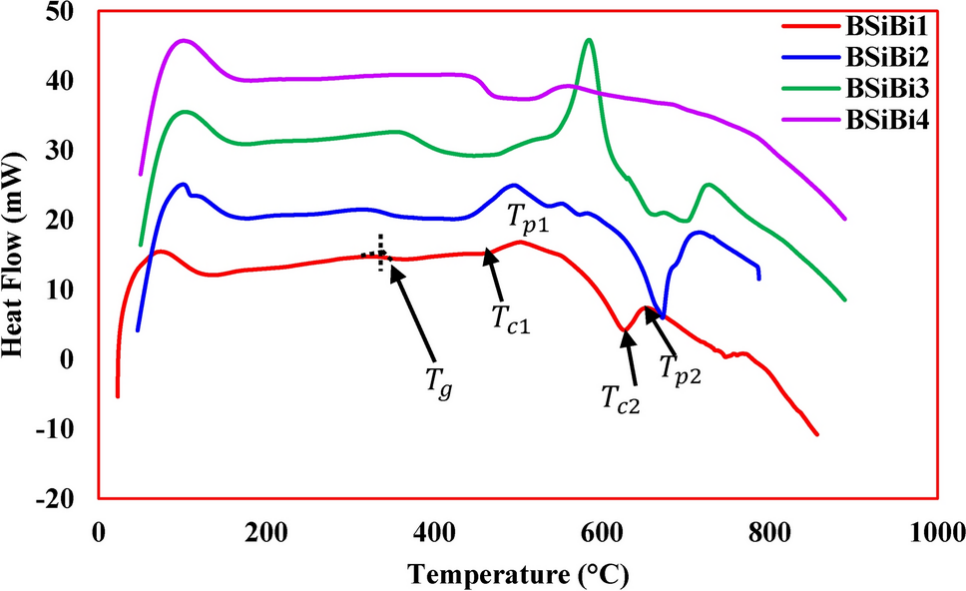
DSC profile of the considered glasses.

Thermal profile, glass transition temperature (${T}_{g}$), onset crystallization temperatures (${T}_{c1}$ & ${T}_{c2}$), and maximum crystallization temperature (${T}_{p1}$ & ${T}_{p2}$), as obtained from DSC (Fig. 6**)** are listed Table 2. The DSC profile of B^3+^ and Bi^3+^ free glass (BSiBi1) shows a broad low intense exothermic peak centered at 508.356 °C, which distinguishes the crystallization temperature. The glass transition temperature of this glass was observed at 341.854 °C. In the considered glasses, as mentioned above, with the addition of both B_2_O_3_ and Bi_2_O_3_, the silicate network is crowded by B-O, Bi-O-Si, Bi-O-B, and B-Bi-Si bonds, in addition to silicate network bonds Si–O. The structural changes in the silicate network that arose from the formation of these bonds, whose number inevitably changes according to the B_2_O_3_ and Bi_2_O_3_ concentrations, and the role of Bi^3+^ in filling the interstice spaces and its entry as a former in the network structure is the main control in changing the behavior of ${T}_{g}$, where it mainly depends on the chemical bond strength and crosslink density of the glass network. As mentioned previously, the ${T}_{g}$ of BSiBi1 glass is 341.854 °C. Upon increase of B_2_O_3_ and Bi_2_O_3_, $\text{B}-\text{O}$ and $\text{Bi}-\text{O}$ bonds, and BO_3_ and BO_4_ units began to form. Hence, the 11.2% reduction in ${T}_{g}$ to become 303.732 °C at 20 mol% of B^3+^ and Bi^3+^ (BSiBi2 glass) was attributed to the presence of BO_3_ groups and the formation of Bi-O bonds, which has a lower bond strength of 102.5 kJ/mol compared to $\text{Si}-\text{O}$ (464 kJ/mol) and B-O (806 kJ/mol). Exceeding 20 mol % barrier of B_2_O_3_ and Bi_2_O_3_ led to significant re-increases again in ${T}_{g}$, which resulted from the richness of the glass network by BO_4_ as shown in Table 2 and overcrowded the glass network by the high bond strength $\text{B}-\text{O}$. In addition to the formation of BO_4_ groups in BSiBi3 and BSiBi4 glasses leading to an increase in the ${T}_{g}$, the non-formation of more $\text{Bi}-\text{O}$ bonds in these glasses as a result of the role of Bi_2_O_3_ in converting BO_3_ to BO_4_ and filling the formed voids more than its participation in the structure is one of the main reasons for increasing the ${T}_{g}$. Finally, all the studied glasses BSiBi1, BSiBi2, BSiBi3, and BSiBi4, show high thermal stability, as all of them showed $\Delta T$ ($\Delta T={T}_{c2}-{T}_{g})$^16,23^ exceeding 100 °C, as listed in Table 2. It is important to note that thermal stability was calculated using the ${T}_{c2}$ rather than the ${T}_{c1}$, as it signifies the temperature range where the glass maintains its amorphous state before significant crystallization begins. ${T}_{c2}$ denotes the peak temperature where crystallization accelerates, marking the onset of the glassy structure’s transformation into a crystalline state.

### Elastic properties

Table 2 shows the behavior of the longitudinal ${v}_{L}$ and shear ${v}_{S}$ velocities and elastic moduli; longitudinal C11, shear C44, bulk B, and Young Y, as well as Poisson’s ratio $\sigma$, microhardness H_UT_, acoustic impedance ${Z}_{i}$, and Debye temperature ${\theta }_{D}$. Elastic properties of glass materials mainly depend on packing density, bond dissociation energy of metal-oxide, cross-linking density and coordination number ^47,48^. The introduction and increase of both B^3+^ and Bi^3+^ ions lead to the formation of the strong bond dissociation energy B-O bonds (498 kJ/mol), high coordination number BiO_6_, and the high network cross-linking units BO_4_ causing an augmentation in longitudinal and shear velocities. Additionally, the effective role of bismuth in filling the glass network interstices, along with its entry into the network, is also a major factor in decreasing the travel time of the waves inside the considered glasses, which means an increase in ultrasonic velocity. It is noted that the augmentation rate in longitudinal and shear velocities is slow up at 20 mol% of B^3+^ and Bi^3+^ ions, which after 20 mol% a high augmentation rate was observed. This behavior arose from the presence of a high ratio of BO_3_ units at 20 mol% of B^3+^ and Bi^3+^ compared to their ratios after 20 mol%, as listed in Table 2. The behavior of density and packing density is fully consistent with the results of longitudinal and shear velocities, and both of them support each other in confirming the rigidity of the studied BSiBi glass series. The behavior of the elastic moduli (longitudinal, shear, bulk, and Young) followed the same pattern, as they all increased with the increase in the concentrations of B^3+^ and Bi^3+^ as shown in Table 2. The augmentation of micro-hardness (H_UT_) and Debye temperature (${\theta }_{D}$) supports the rigidity of the considered glasses and the results of the elastic moduli. The observed reduction in the Poison ratio ($\sigma$), as listed in Table 2, reflects the increase in the cross-linking density as a result of the overcrowded of the glass network by Bi^3+^ ions and the formation of BO_4_ units confirming the interpretation of ultrasonic velocities and elastic moduli. The augmentation of acoustic impedance (${{\varvec{Z}}}_{{\varvec{i}}}$), which refers to a material’s resistance to ultrasonic wave propagation attributed to the formation of BO’s confirming the compactness of the considered network.

### Optical properties

**Fig. 7:**
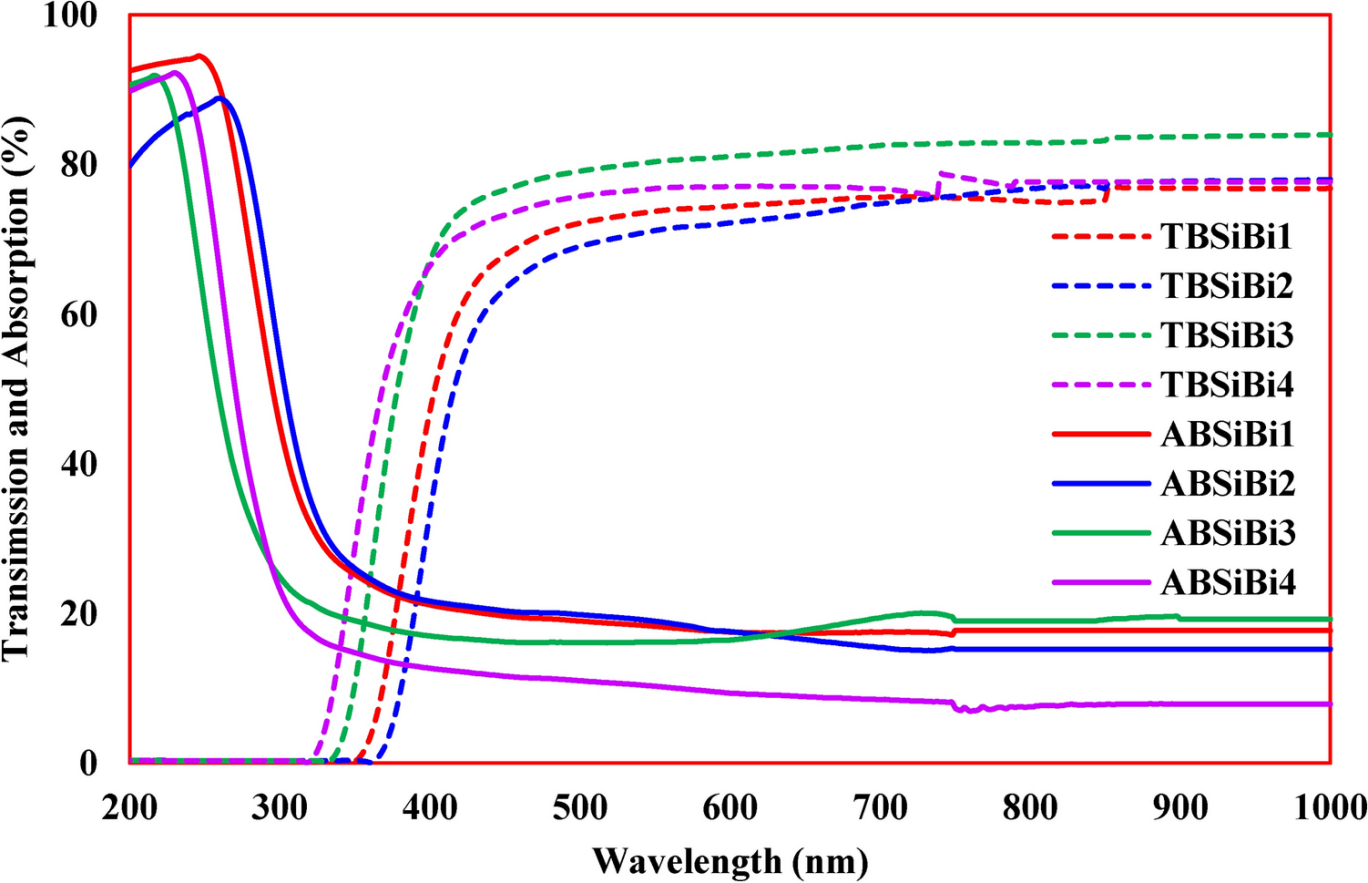
Optical transmission (dash lines) and absorption (solid lines) spectra of the considered BSiBi glasses.

The considered glasses were characterized by high visible light transparency, as previously discussed in the ocular description (Fig. 1), and as is evident in the transmittance (dash lines) and absorption (solid lines) curves in Fig. 7. On the other hand, it has been observed that the studied glasses are very effective in the full absorption of the UV region. In general, a blue shift was observed in the cut-off wavelength, except for glass containing 20 mol% of B^3+^ and Bi^3+^ ions (BSiBi3), which has a slightly red shift. The blue shift in the cut-off wavelength was attributed to the existence of more concentrations of BO_4_ groups compared to BO_3_, while for BSiBi3 glass; the concentrations of BO_3_ were significantly higher than BO_4_, as listed in Table 2. It is noteworthy that there are some perturbations in the optical spectra around 750 nm, attributed to detector jumps.

**Fig. 8:**
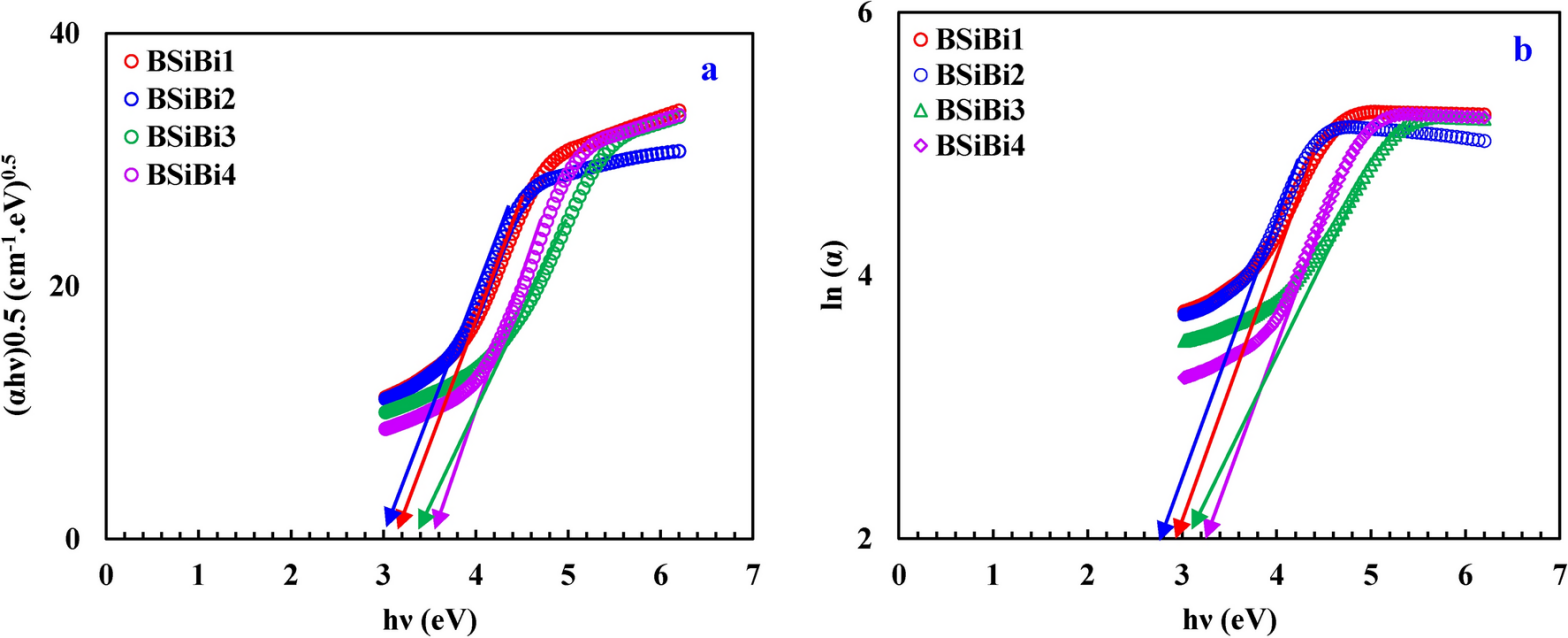
(**a**) Tauc plot to deduce optical band gap and (**b**) relation between of $\text{ln}\alpha$ and $h\nu$ to obtain Urbach energy.

Optical band gap (${E}_{g}$) and Urbach energy ($\Delta E$) are estimated from Fig. 8a and b based on Eqs. (23 and 24)^49–51^, and listed in Table 2. The reciprocal of the obtained values from Fig. 8b was taken to estimate the Urbach energy. Generally, an augmentation in the optical band gap and reduction in Urbach energy were observed except for BSiBi2 glass. The augmentation of band gap and reduction of Urbach energy attributed to the formation of BO_4_ units, which the reduction of band gap and increase of Urbach energy for the BSiBi2 arose as a result of growth of BO_3_ units at the expense of BO_4_ ones.

23 $$\alpha h\nu =\beta {\left(h\nu -{E}_{g}\right)}^{n}$$

24 $$\alpha = \beta e^{{{\raise0.7ex\hbox{${hv}$} \!\mathord{\left/ {\vphantom {{hv} {\Delta E}}}\right.\kern-0pt} \!\lower0.7ex\hbox{${\Delta E}$}}}}$$

where, $\alpha$, $h\nu$, and $\beta$ are the absorption coefficient, the incident photon energy, and the band tailing parameter. On the other hand, the $n$ refers to the type of electronic transition and taken to be 2 due to the most popular transition in the amorphous materials is the indirect one.

### Shielding properties

**Fig. 9:**
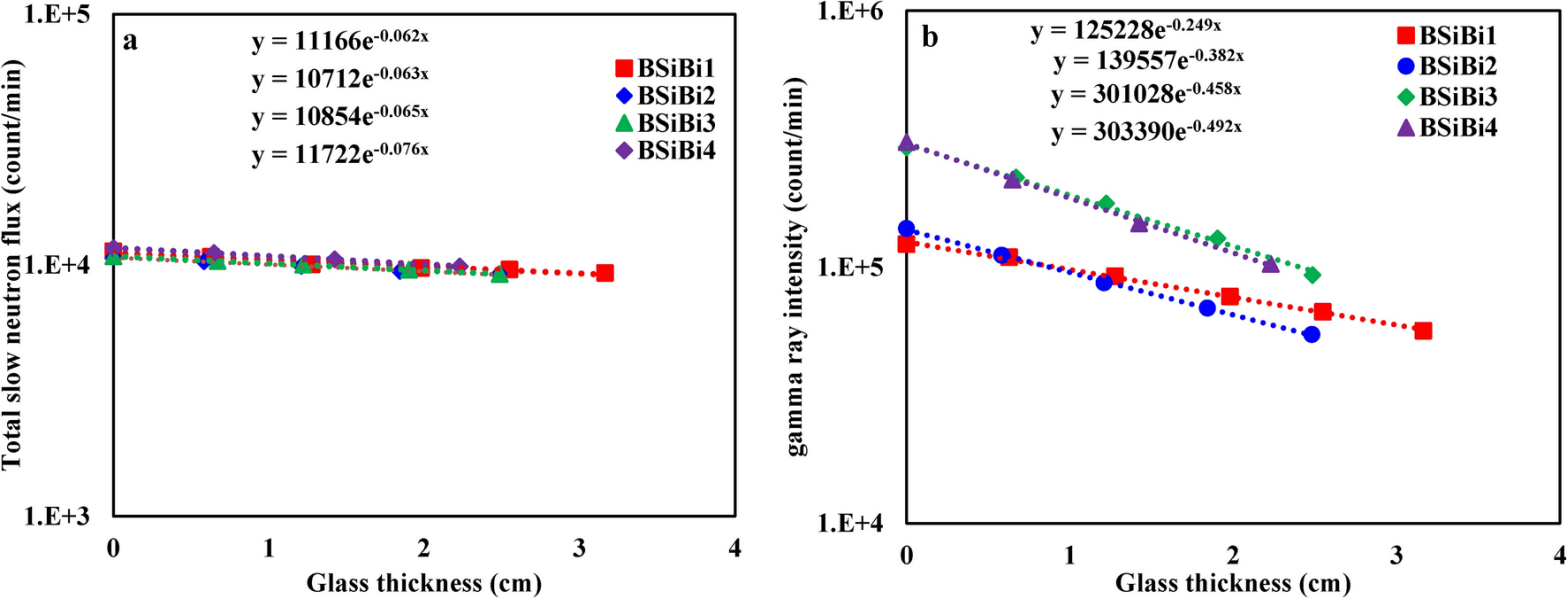
Neutron fluxes and gamma ray intensities as a function of glass thickness: (**a**) total slow neutrons and (**b**) gamma ray energy of 661.64 keV as examples.

To examine the attenuation efficiency of total slow neutrons, slow neutrons, and gamma rays of different energies for the studied glass series, the transmitted neutron fluxes and the intensity of gamma rays through the considered glass materials were measured and plotted as a function of the variation in the glass thickness according to Lambert’s law, as shown in Fig. 9 (examples). These relations were then used to derive the macroscopic cross section of the total slow and slow neutrons and the linear attenuation coefficient of gamma rays.

#### Neutron shielding

**Fig. 10:**
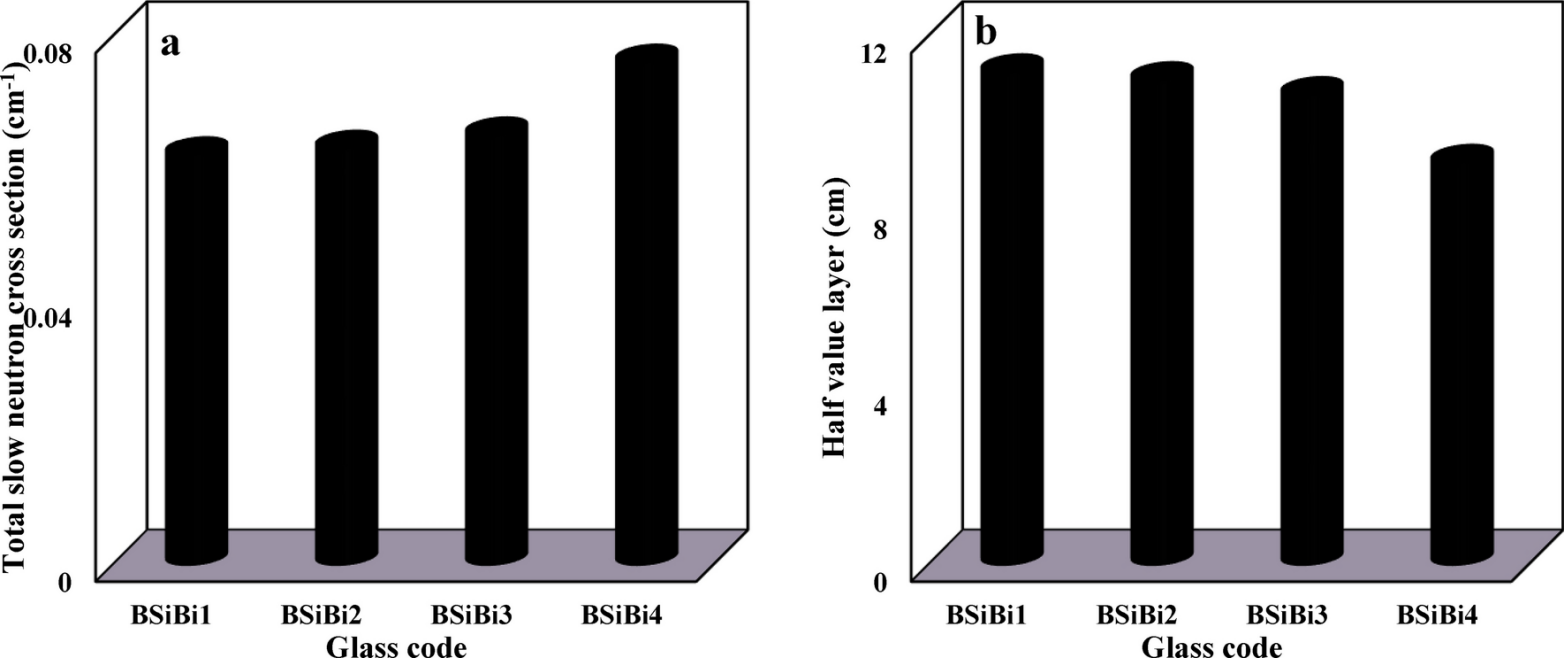
(**a**) The macroscoptic cross-section and (**b**) the half-value layer of the total slow neutrons of the considered BSiBi glass series.

**Fig. 11:**
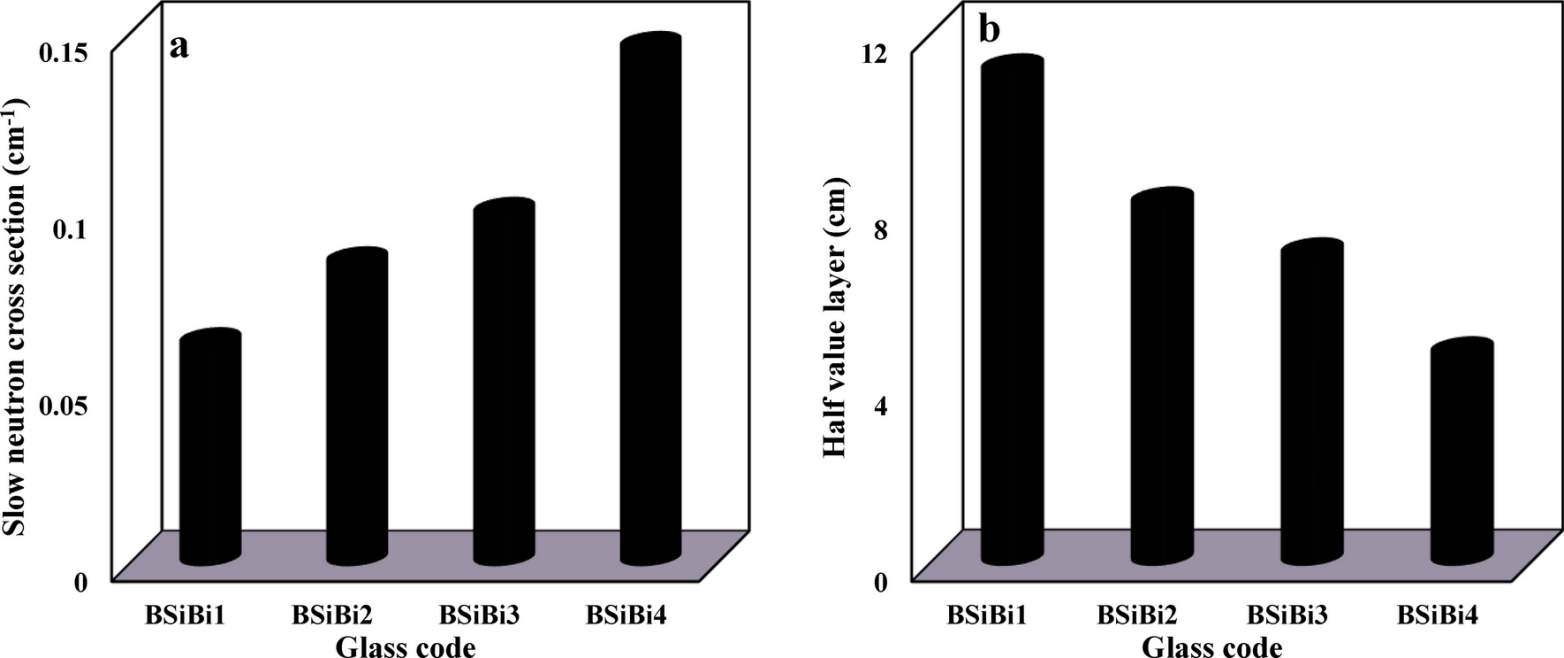
(**a**) The macroscoptic cross-section and (**b**) the half-value layer of the slow neutrons of the considered BSiBi glass series.

The estimated values of the total slow and slow neutrons’ macroscopic cross-section and the equivalent half-value layers for each of them are shown in Figs. 10 and 11, respctively. First, as we explained previously, the total slow neutrons are the result of slow neutrons emerging from the source (primary slow neutrons), in addition to those emitted from the source as fast ones and decelerated as a result of their interaction with the studied glass constituents (secondary slow neutrons). Hence, the attenuation of the total slow neutrons is a dual process: slowing down the fast neutrons and absorbing the slow ones (primary and secondary neutrons simultaneously). According to Hila et al., Si, Na, O, B, and Bi nuclei have mass removal cross sections of fast neutrons that were emitted from ^241^Am-Be of 0.03070, 0.03649, 0.02709, 0.05627, and 0.00926 cm^2^/gm, respectively ^52^. Hence, since B and Bi nuclei together have higher mass removal cross sections of fast neutrons than Na, increasing their concentrations at the expense of Na enhances the ability of the considered BSiBi glass series to slow down fast neutrons. On the other hand, boron nuclei, without competition from the other constituents of the studied BSiBi glass series, has the highest absorption cross-section of slow neutrons ^12,53,54^, leading to an increase in the ability of the glass to attenuate slow neutrons. Consequently, as was clearly shown in Fig. 10a and 11a, the values of the macroscopic cross-section of the total slow neutrons increased with increasing concentrations of B and Bi nuclei simultaneously due to their higher fast neutron removal cross-section compared to Na and the highest slow neutron absorption cross-section of B.

The total slow neutrons attenuation efficiency was enhanced by 22.6% with the growth of B and Bi nuclei concentrations, while attenuation of the slow neutrons was improved by 135.5% as a result of the augmentation of B nuclei concentrations. As shown in Figs. 10b and 11b, a monotonous reduction was observed in the half-value layers, which is the parameter responsible for clarifying the required thickness to reduce half of the emitted radiation, from 11.180 and 11.180 at 10 mol% of B and Bi to 9.120 and 4.748 cm at 40 mol% of B and Bi. The observed reduction in the half-value layer confirms the improvement in the efficiency of the studied glass shield in attenuating total slow and slow neutrons.

#### Gamma ray shielding

**Fig. 12:**
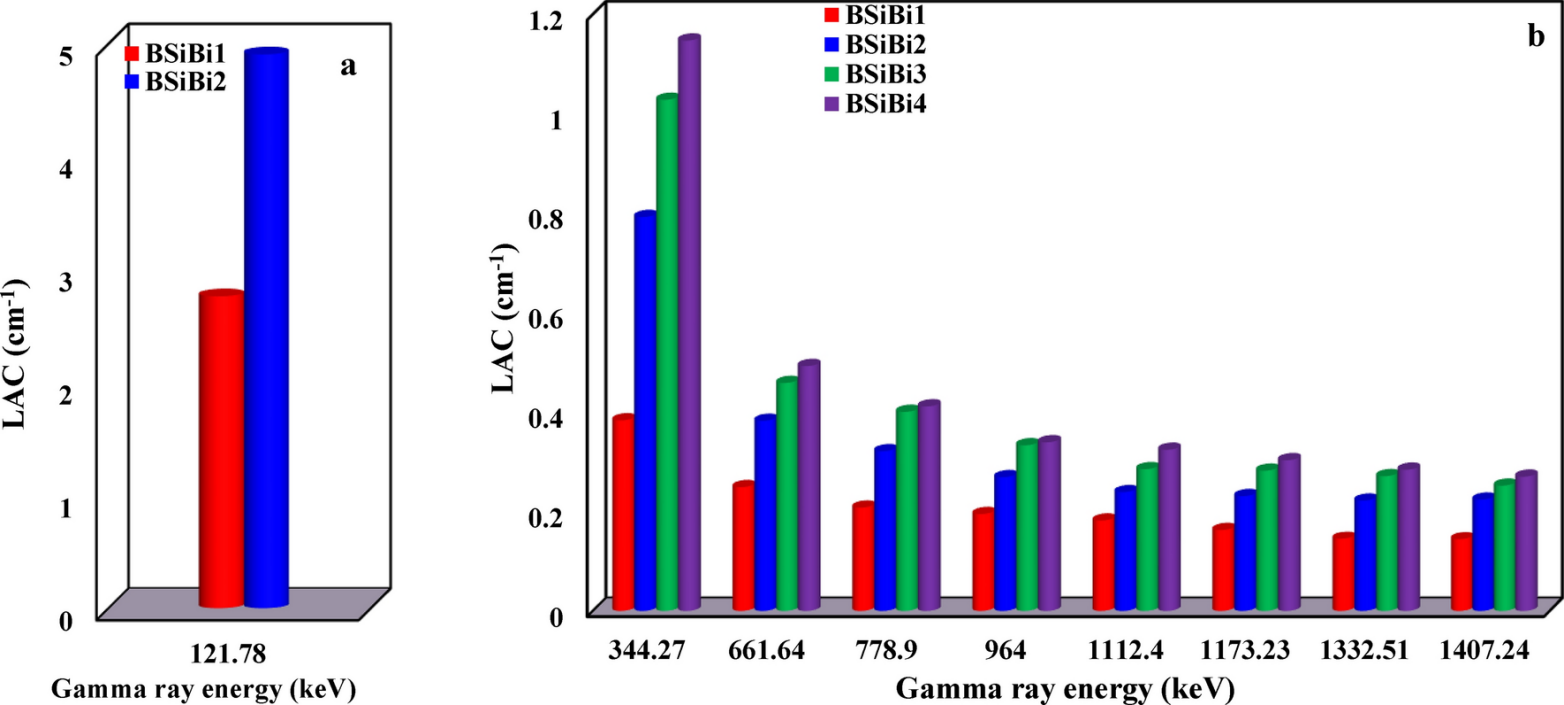
Linear attenuation coeffecients of the considered BSiBi glass series at energies of (**a**) 121.78 keV and (**b**) 344.27, 661.64, 778.9, 964, 1112.4, 1332.51, 1407.24 keV of gamma rays.

**Fig. 13:**
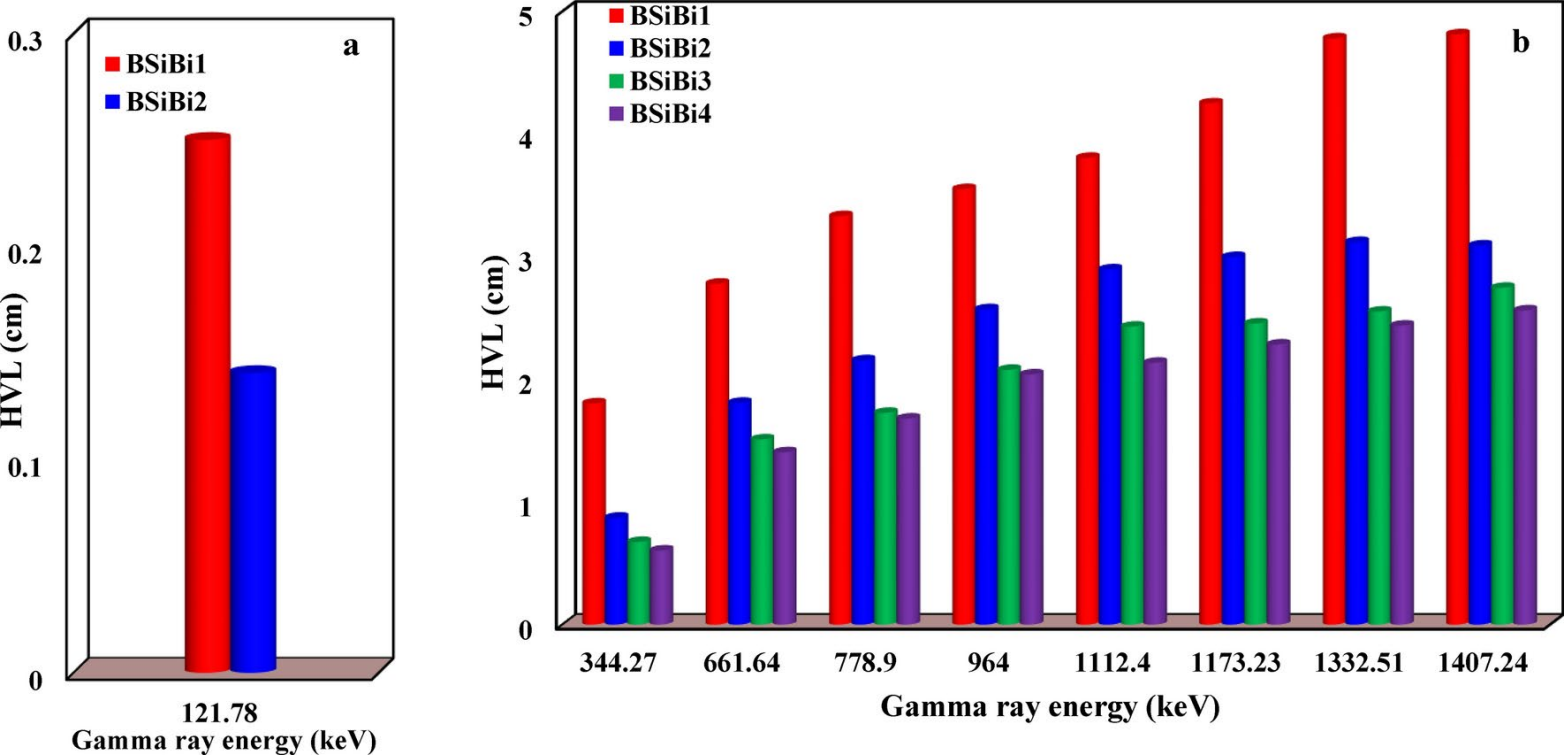
Half value layer of the considered BSiBi glass series at gamma ray energies of (**a**) 121.78 keV and (**b**) 344.27, 661.64, 778.9, 964, 1112.4, 1332.51, 1407.24 keV.

Figures 12 and 13 show the study of the attenuation ability of the studied BSiBi glass series through examination of the variation in the values of the linear attenuation coefficient and half-value layer with increasing bismuth concentrations at different gamma energies. First, from Figs. 12a and 13a, it was noted that the linear attenuation coefficient (LAC, $\mu$) and half-value layer (HVL) of the 121.78 keV were studied for BSiBi1 and BSiBi2 glass only, because this low energy had a high interaction probability with the considered glass with increasing concentrations of bismuth, and it had completely attenuated at a concentration of 20 mol% of Bi. In Figs. 12b and 13b, a monotonous increase in the linear attenuation coefficient and a decrease in half-value layer values were observed with increasing Bi-concentration at the various studied gamma rays’ energies due to its richness in electrons, which increased the possibility of gamma ray interaction with the considered BSiBi glass medium. Based on the measured linear attenuation coefficient values, the mass attenuation coefficient was estimated experimentally (${\sigma }_{exp.}$) and compared to its theoretically counterpart (${\sigma }_{the.}$), which is calculated using XCOM software, as listed in Table 3. Firstly, a good convergence was observed between the measured and theoretical results for gamma rays $\ge$ 661.64 keV, which indicates the accuracy of the measured results. However, there is a fairly large discrepancy at energies 121.78 and 344.27 keV, which arose due to the very high attenuation of those energies through the photoelectric effect interaction ^55^, whose cross-section (${\sigma }_{ph}$) strongly depends on the atomic number (${\sigma }_{ph}\propto {Z}^{3.6-5.3}$) ^56,57^. On the other hand, with the increase in gamma ray energy, a reduction was observed in the rate of increase of the mass attenuation coefficient with increasing Bi concentrations, which occurred as a result of the dominance of the Compton effect, in which the dependence of its cross-section (${\sigma }_{C}$) on the atomic number is very weak (${\sigma }_{ph}\propto Z$) ^56,57^.

**Table 3.** Expermintal and theoretical mass attenuation coeffecient of the considered BSiBi glass.

Gamma ray energy (keV)	MAC (cm^2^/gm)	Glass Code			
		BSiBi1	BSiBi2	BSiBi3	BSiBi4
121.78	${\sigma }_{exp.}$	0.796 $\pm$ 0.008	1.155 $\pm$ 0.011	–	–
	${\sigma }_{the.}$	0.428	0.715	–	–
344.27	${\sigma }_{exp.}$	0.110 $\pm$ 0.004	0.187 $\pm$ 0.004	0.212 ± 0.007	0.211 ± 0.006
	${\sigma }_{the.}$	0.116	0.134	0.151	0.169
661.64	${\sigma }_{exp.}$	0.072 $\pm$ 0.008	0.090 $\pm$ 0.006	0.095 ± 0.004	0.091 ± 0.004
	${\sigma }_{the.}$	0.078	0.081	0.084	0.087
778.9	${\sigma }_{exp.}$	0.060 $\pm$ 0.013	0.076 $\pm$ 0.003	0.083 ± 0.005	0.076 ± 0.004
	${\sigma }_{the.}$	0.072	0.073	0.075	0.077
964	${\sigma }_{exp.}$	0.056 $\pm$ 0.022	0.063 $\pm$ 0.020	0.069 ± 0.020	0.062 ± 0.019
	${\sigma }_{the.}$	0.064	0.065	0.066	0.067
1112.4	${\sigma }_{exp.}$	0.052 $\pm$ 0.010	0.056 $\pm$ 0.019	0.059 ± 0.022	0.060 ± 0.009
	${\sigma }_{the.}$	0.059	0.060	0.060	0.061
1173.23	${\sigma }_{exp.}$	0.047 $\pm$ 0.013	0.054 $\pm$ 0.009	0.058 ± 0.008	0.056 ± 0.005
	${\sigma }_{the.}$	0.058	0.058	0.058	0.059
1332.51	${\sigma }_{exp.}$	0.042 $\pm$ 0.005	0.052 $\pm$ 0.017	0.056 ± 0.011	0.052 ± 0.009
	${\sigma }_{the.}$	0.054	0.054	0.054	0.055
1407.24	${\sigma }_{exp.}$	0.041 $\pm$ 0.008	0.053 $\pm$ 0.005	0.052 ± 0.018	0.050 ± 0.019
	${\sigma }_{the.}$	0.052	0.053	0.053	0.053

## Conclusion

A successful attempt to produce silicate glass reinforced with different ions, B^3+^ and Bi^3+^, to fabricate an efficient radiation shield for total slow and slow neutrons and gamma rays of different energies was carried out. The structural examination revealed the formation of $\text{Bi}-\text{O}-\text{Si}$, $\text{Bi}-\text{O}-\text{B}$, and $\text{B}-\text{Bi}-\text{Si}$ bonds and borosilicate glass network bonds as a result of increasing the B^3+^ and Bi^3+^ concentrations. Additionally, the ratio of bridging oxygen units grows within the considered BSiBi glass series except at 20 mol% of B_2_O_3_ and Bi_2_O_3_. The structural changes resulting from the addition of B^3+^ and Bi^3+^ ions resulted in a significant increase in the elastic moduli and hardness of the studied BSiBi glass. The elastic moduli, longitudinal, shear, bulk, and Young were improved by 107.9, 138.8, 88.5, and 130.3%, respectively, while the hardness was improved by 191.7% at 40 mol% of Bi and B. In the visible light region, the studied BSiBi glass series had a high transparency that exceeded 70%, and it also had a high ability to completely block ultraviolet radiation. Thermally, the BSiBi glass series has high thermal stability, and the thermal properties’ fluctuations of the glass network were in perfect tune with the structural changes. The optical gap energy of all the considered BSiBi glass series exceeded 3 eV, indicating their insulating nature, while the behavior of the Urbach energy corresponded to the disorder within the glass network. From a shielding perspective, increasing boron and bismuth concentrations simultaneously enhanced the glass’s ability to attenuate the total slow neutrons. While increasing boron enhanced the attenuation of slow neutrons, bismuth increased the attenuation of gamma rays of different energies. Additionally, it is observed that the low energy gamma rays at 128.78 keV was completely attenuated at 20 mol% of Bi. The attenuation performance of total slow neutrons, slow neutrons and gamma rays was improved by 22.6, 135.5, and 73.8 $-$ 199.5%. Based on the conclusions reached regarding the high optical transparency, thermal stability, elasticity, and attenuation performance for both total slow and slow neutrons and gamma rays, the BSiBi4 glass (40 mol% of B_2_O_3_ and Bi_2_O_3_) is considered a suitable effective radiation shield in various nuclear domains.

## Data Availability

The datasets used and/or analysed during the current study available from the corresponding author on reasonable request.
